# Inflammatory Hematological Indices in Heart Failure with Preserved Ejection Fraction: Associations with Disease Severity and 12-Month Outcomes

**DOI:** 10.3390/biomedicines14102301

**Published:** 2026-10-10

**Authors:** Cristian Maghet, Diana-Aurora Arnăutu, Sergiu-Florin Arnautu, Bogdan-Flaviu Buz, Mirela-Cleopatra Tomescu, Tudor Rareș Olariu

**Affiliations:** 1 Doctoral School, Victor Babeș University of Medicine and Pharmacy, 300041 Timișoara, Romania; cristian.maghet@umft.ro; 2 Multidisciplinary Heart Research Center, Victor Babeș University of Medicine and Pharmacy, 300041 Timișoara, Romania; 3 Center for Diagnosis and Study of Parasitic Diseases, Department of Infectious Diseases, Victor Babeș University of Medicine and Pharmacy, 300041 Timișoara, Romania; rolariu@umft.ro; 4 Multidisciplinary Heart Research Center, Department of Internal Medicine I, Faculty of Medicine, “Victor Babes” University of Medicine and Pharmacy, 2nd Eftimie Murgu Square, 340001 Timisoara, Romania; flaviu-bogdan.buz@umft.ro (B.-F.B.); tomescu.mirela@umft.ro (M.-C.T.); 5 Cardiology Clinics, Timisoara Clinical Municipal Emergency Hospital, Bd. Revolutiei din 1989, No. 12, 300041 Timisoara, Romania; 6 Centre for Cognitive Research in Neuropsychiatric Pathology (Neuro-Psy-Cog), Department of Neurosciences, “Victor Babes” University of Medicine and Pharmacy from Timisoara, E. Murgu Sq., No. 2, 300041 Timisoara, Romania; 7 Department of Infectious Diseases, Victor Babeș University of Medicine and Pharmacy, 300041 Timișoara, Romania; 8 Clinical Laboratory, Municipal Clinical Emergency Hospital, 300041 Timisoara, Romania; 9 Patogen Preventia, 300124 Timisoara, Romania

**Keywords:** HFpEF, cardiometabolic inflammation, microvascular dysfunction, immune dysregulation, NLR, RDW, PLR, biomarkers, risk stratification, prognosis

## Abstract

**Background/Objectives**: Heart failure with preserved ejection fraction (HFpEF) is increasingly recognized as a systemic inflammatory syndrome with limited diagnostic and prognostic tools applicable in routine practice. Hematological indices derived from complete blood count, including neutrophil-to-lymphocyte ratio (NLR), red cell distribution width (RDW), and platelet-to-lymphocyte ratio (PLR), have emerged as potential markers of systemic inflammation and cardiovascular risk. This study aimed to compare these indices between patients with HFpEF and individuals without heart failure and to examine their associations with disease severity and 12-month clinical outcomes. **Methods**: In this retrospective observational study, 221 patients with confirmed HFpEF were compared with 143 control subjects without heart failure. NLR, RDW, and PLR were calculated from routine laboratory tests. Associations with echocardiographic parameters, NT-proBNP levels, and clinical outcomes were analyzed. Patients were followed for 12 months to assess major adverse cardiovascular events (MACEs), all-cause mortality, and heart failure-related rehospitalization. Multivariable Cox regression models were used to evaluate independent prognostic value. **Results**: Patients with HFpEF exhibited significantly higher NLR (3.98 ± 0.98 vs. 2.72 ± 0.81), RDW (14.62 ± 1.09% vs. 13.48 ± 0.92%), and PLR (152.4 ± 38.7 vs. 131.9 ± 29.6) compared with controls (all *p* < 0.001). NLR and RDW correlated with NT-proBNP, E/e′ ratio, left atrial volume index, and NYHA class. Higher tertiles of NLR and RDW were associated with progressively increased rates of MACEs and all-cause mortality, with statistically significant trends across tertiles (all p-trend ≤ 0.008). In multivariable analysis, NLR (HR 1.28, *p* = 0.001) and RDW (HR 1.32, *p* < 0.001) remained independently associated with 12-month MACEs, while PLR showed borderline significance. However, ROC analysis demonstrated limited discrimination, with AUC values ranging from approximately 0.55 to 0.61, indicating that none of the three indices reliably discriminated between patients with and without MACEs when considered individually. **Conclusions**: NLR and RDW were elevated in patients with HFpEF and were independently associated with disease severity and 12-month MACEs. However, all three indices showed limited individual discrimination and should not be considered standalone prognostic tools. Their potential clinical value, if any, requires evaluation as part of validated multivariable prediction models.

## 1. Introduction

Heart failure with preserved ejection fraction (HFpEF) has become one of the most challenging entities in contemporary cardiology [1]. Once considered a relatively benign form of heart failure, HFpEF is now recognized as a complex clinical syndrome, deeply rooted in systemic inflammation, metabolic dysregulation, endothelial dysfunction, and multi-organ interaction [2]. Prevalence continues to rise, driven by aging populations, obesity, hypertension, diabetes, and the global epidemic of metabolic syndrome, making HFpEF a dominant phenotype among heart failure admissions [3]. Despite its rising prevalence, HFpEF remains challenging to diagnose, and current risk stratification tools are still limited, particularly in routine clinical practice [4,5].

In recent years, attention has shifted toward inflammation as a central mechanism in HFpEF pathogenesis [6]. Low-grade systemic inflammation contributes to microvascular rarefaction, increased myocardial stiffness, impaired relaxation, and altered energetic metabolism [7]. As a result, standard biomarkers such as natriuretic peptides, although useful, do not fully capture the inflammatory and oxidative milieu characteristic of HFpEF [8]. In this context, simple hematological indices derived from routine complete blood count (CBC) have gained increasing interest as potential markers of both systemic inflammation and cardiovascular risk [9].

The neutrophil-to-lymphocyte ratio (NLR) is one of the most investigated inflammatory markers in cardiovascular diseases [10]. Neutrophilia reflects an activated inflammatory response, while lymphopenia is often associated with physiological stress and impaired immune regulation [11]. Consequently, NLR integrates two complementary pathways and has been linked to poor outcomes in coronary syndromes, heart failure exacerbations, arrhythmias, and even long-term mortality [12]. In HFpEF specifically, several studies report that elevated NLR correlates with disease severity, functional limitation, and increased risk of hospitalization [13].

The red cell distribution width (RDW), a measure of variability in erythrocyte volume, was initially regarded as a hematologic parameter with relevance only in anemia [14]. However, emerging evidence shows that elevated RDW mirrors oxidative stress, impaired erythropoiesis, chronic inflammation, and nutritional imbalance, elements that commonly coexist in HFpEF patients [15]. Higher RDW values have been consistently associated with increased mortality and adverse outcomes across all spectrums of heart failure [16].

The platelet-to-lymphocyte ratio (PLR) adds another layer by combining thrombocytic activation with immune dysregulation [17]. Since platelets play a significant role in inflammation and microvascular dysfunction, PLR may provide valuable insights into the pro-inflammatory and pro-thrombotic environment that characterizes HFpEF [18].

One of the advantages of NLR, RDW, and PLR is their universal availability: they are inexpensive, reproducible, easy to calculate, and routinely obtained in clinical practice. This makes them appealing candidates for large scale risk stratification, especially in resource-limited settings or retrospective research designs [19]. Yet, despite their promise, their role in the specific context of HFpEF, particularly in differentiating these patients from individuals without heart failure and in predicting subsequent adverse events, remains insufficiently defined [20].

Therefore, the present retrospective study aimed to determine whether NLR, RDW, and PLR differed between patients with HFpEF and individuals without heart failure and to examine their associations with markers of disease severity and subsequent clinical outcomes. Because no diagnostic classification analysis was performed, the study was not designed to establish diagnostic thresholds or evaluate these indices as diagnostic tests for HFpEF.

## 2. Materials and Methods

### 2.1. Study Design and Setting

This retrospective observational study was conducted at the Timișoara Municipal Emergency Clinical Hospital, a tertiary center serving a diverse population of patients with cardiovascular and metabolic diseases in Western Romania. The study covered a period of three consecutive years, from January 2022 to December 2024, and included both hospitalized patients and individuals evaluated in the cardiology and internal medicine outpatient departments.

### 2.2. Study Population

During the study period, 364 adult patients who met the eligibility criteria and had complete blood count, clinical, and echocardiographic data available were included in the study. Of these, 221 fulfilled the diagnostic criteria for heart failure with preserved ejection fraction (HFpEF) and constituted the HFpEF cohort. The diagnosis was established according to contemporary European Society of Cardiology (ESC) guidelines and required: (1) a preserved left ventricular ejection fraction (LVEF ≥ 50%); (2) symptoms and/or signs of heart failure; (3) elevated plasma N-terminal pro-B-type natriuretic peptide (NT-proBNP) levels; and (4) echocardiographic evidence of diastolic dysfunction or relevant structural cardiac abnormalities, such as an increased left atrial volume index or left ventricular hypertrophy [21,22].

Patients were eligible regardless of whether they had been evaluated as inpatients for suspected cardiac decompensation or as outpatients undergoing chronic cardiologic assessment, provided that the diagnostic criteria and the required clinical, laboratory, and echocardiographic data were adequately documented.

The remaining 143 eligible patients had no evidence of heart failure and constituted the control group. These participants were evaluated at the same institution and during the same study period, either during hospitalization for non-acute cardiovascular or metabolic conditions or during routine outpatient follow-up. Eligibility for the control group required no history of heart failure, a preserved LVEF, normal or near-normal natriuretic peptide levels, and no symptoms or clinical signs suggestive of cardiac dysfunction. No individual, frequency, propensity-score, or other formal matching procedure was performed. Age and cardiovascular risk factors were not used as matching variables; therefore, any similarities in these characteristics between the HFpEF and control groups reflected the observed composition of the cohorts rather than matching by study design.

### 2.3. Inclusion and Exclusion Criteria

Eligible patients were adults aged 18 years or older, who had undergone a CBC and a transthoracic echocardiogram during the same clinical encounter. Their medical records had to contain sufficient clinical, laboratory, and echocardiographic data to confirm or exclude HFpEF and to characterize comorbidities and medication exposures.

To avoid the confounding influence of inflammatory or hematologic conditions, several exclusion criteria were applied. Patients were excluded if they had documented acute or chronic infections in the four weeks preceding evaluation, active malignancies or were receiving chemotherapy, hematologic cancers, or known bone marrow disorders. Additional exclusions included autoimmune diseases requiring systemic immunosuppression, recent acute coronary syndrome or stroke within the last three months, and end-stage renal disease requiring dialysis. Patients on long-term systemic corticosteroid therapy were also excluded due to its potential to significantly alter leukocyte and platelet dynamics.

### 2.4. Data Collection

For all eligible individuals, data were extracted from electronic hospital records, including demographic variables such as age and sex, as well as cardiovascular risk factors and comorbidities such as hypertension, diabetes mellitus, obesity, chronic kidney disease, or documented ischemic heart disease. Baseline medication use was determined from active prescriptions documented at the index evaluation. The treatment classes recorded included diuretics, beta-blockers, angiotensin-converting enzyme inhibitors or angiotensin receptor blockers (ACEIs/ARBs), angiotensin receptor–neprilysin inhibitors (ARNIs), mineralocorticoid receptor antagonists (MRAs), sodium–glucose cotransporter 2 (SGLT2) inhibitors, and other glucose-lowering medications. Because combination therapy was common, medication categories were not mutually exclusive, and individual patients could be included in more than one treatment class.

Laboratory values included CBC parameters, neutrophil, lymphocyte, and platelet counts, as well as RDW. These values were used to calculate the hematological inflammatory indices of interest: NLR, PLR, and the RDW coefficient of variation.

When documented, natriuretic peptide levels, renal and hepatic function tests, and echocardiographic measurements were also retrieved.

### 2.5. Clinical Outcomes and Follow-Up

Patients included in the HFpEF group were followed for 12 months after the index evaluation. Clinical outcomes were ascertained from electronic medical records, hospital readmission records, and outpatient follow-up documentation. The outcomes of interest were major adverse cardiovascular events (MACEs), all-cause mortality, and heart failure-related rehospitalization. The MACE composite comprised cardiovascular death, non-fatal myocardial infarction, and non-fatal stroke. Heart failure-related rehospitalization was defined as an unplanned hospital admission primarily attributable to worsening signs or symptoms of heart failure requiring inpatient treatment. This outcome was analyzed separately and was not included in the MACE composite. For each outcome, patients were counted once based on the occurrence of at least one qualifying event during follow-up. In the time-to-event analyses, the time to the first qualifying event was considered, and patients without an event were censored at 12 months.

### 2.6. Statistical Analyses

Statistical analyses were performed using GraphPad Prism version 10.0 (GraphPad Software, San Diego, CA, USA) for data visualization and the majority of descriptive and inferential analyses. The distribution of continuous variables was assessed using the Shapiro–Wilk test. Normally distributed variables are presented as mean ± standard deviation (SD), while non-normally distributed variables are expressed as median with interquartile range (IQR). Categorical variables are reported as absolute numbers and percentages.

Comparisons between the HFpEF and control groups were conducted using unpaired Student’s t-test for normally distributed continuous variables and the Mann–Whitney U test for non-normally distributed data. Differences in categorical variables were evaluated using the χ^2^ test or Fisher’s exact test, as appropriate. Associations between hematological inflammatory indices (NLR, RDW, PLR) and clinical, biochemical, or echocardiographic parameters were examined using Pearson’s correlation coefficient or Spearman’s rank correlation, depending on data distribution.

Time-to-event analyses were performed using Kaplan–Meier survival curves, with between-group differences assessed by the log-rank test. Receiver operating characteristic (ROC) curve analysis was used to evaluate the discriminative ability of inflammatory indices for predicting 12-month major adverse cardiovascular events.

Statistical analyses not fully supported in GraphPad Prism, including trend analyses across tertiles and Cox regression analyses, were performed using IBM SPSS Statistics version 26.0 (IBM Corp., Armonk, NY, USA). All statistical tests were two-sided, and a *p*-value < 0.05 was considered statistically significant.

Univariable Cox proportional hazards analyses were initially performed to examine the association between each candidate predictor and 12-month MACEs. Variables included in the multivariable model were prespecified based on their established clinical relevance in HFpEF and their potential association with adverse cardiovascular outcomes, rather than selected solely according to statistical significance in the univariable analyses. The final multivariable model included NLR, RDW, PLR, age, sex, hypertension, type 2 diabetes mellitus, chronic kidney disease, NT-proBNP, and the E/e′ ratio. All covariates were entered simultaneously into the model. The proportional hazards assumption was evaluated using log-minus-log survival plots and by testing interactions between each covariate and follow-up time. No relevant violations of the proportional hazards assumption were identified. Results are reported as hazard ratios (HRs) with 95% confidence intervals (CIs).

Baseline medication use was summarized descriptively. Individual medication classes were not incorporated into the primary multivariable Cox model because only 62 MACEs occurred during follow-up, and the inclusion of several additional treatment variables would have further increased the risk of model overfitting. In addition, treatment allocation was not randomized and was influenced by clinical indication, disease severity, and comorbidity burden, creating the potential for confounding by indication.

### 2.7. Ethical Approval

The study protocol was reviewed and approved prior to study initiation by the Ethical Committee for Scientific Research of the “Victor Babeș” University of Medicine and Pharmacy Timișoara, Romania (Approval No. 10/10 March 2023). The Ethical Committee operates in accordance with Article 167 of Law no. 95/2006, Article 28, Chapter VIII of Order 904/2006; the EU Good Clinical Practice Directive 2005/28/EC; the International Conference on Harmonisation of Technical Requirements for Registration of Pharmaceuticals for Human Use (ICH-GCP); and the principles outlined in the Declaration of Helsinki regarding biomedical research involving human subjects.

Given the retrospective observational nature of the study and the use of anonymized clinical data, patient confidentiality and data protection were strictly maintained throughout all stages of data collection and analysis.

## 3. Results

The study cohort comprised 221 patients with HFpEF and 143 control participants. The mean age was 70.8 ± 8.5 years in the HFpEF group and 69.1 ± 9.1 years in the control group. Female sex was slightly more prevalent in both groups (60.6% vs. 58.0%, respectively). Baseline demographic, clinical, and echocardiographic characteristics are summarized in Table 1. Overall, the HFpEF and control groups were broadly comparable with respect to age, sex distribution, body mass index, and the prevalence of major cardiometabolic comorbidities, including hypertension, type 2 diabetes mellitus, dyslipidemia, and chronic kidney disease. Resting hemodynamic parameters, such as systolic blood pressure and heart rate, as well as left ventricular ejection fraction, were also similar between groups. In contrast, patients with HFpEF exhibited clear differences in markers of diastolic dysfunction and cardiac remodeling, including significantly higher E/e′ ratios and left atrial volume index, alongside markedly elevated circulating NT-proBNP levels, consistent with increased filling pressures and myocardial stress. As expected, a substantial proportion of HFpEF patients were classified as NYHA functional class III-IV, reflecting greater symptom burden in this group.

Baseline pharmacological treatment in the HFpEF cohort is also presented in Table 1. At the index evaluation, 158 patients (71.5%) were receiving diuretics, 139 (62.9%) beta-blockers, and 116 (52.5%) an ACEI or ARB. ARNI therapy was documented in 22 patients (10.0%), while 72 patients (32.6%) were receiving a mineralocorticoid receptor antagonist. SGLT2 inhibitors were used by 51 patients (23.1%), and 66 patients (29.9%) were receiving other glucose-lowering medications. As medication categories were not mutually exclusive, patients receiving combination therapy were counted in each applicable treatment class.

Hematological parameters differed between patients with HFpEF and control participants (Table 2). NLR, PLR, and RDW were significantly higher in the HFpEF group than in the control group (all *p* < 0.001). Patients with HFpEF also had slightly lower hemoglobin levels (12.6 ± 1.2 vs. 12.9 ± 1.1 g/dL; *p* = 0.04). Platelet counts did not differ significantly between the two groups (235 ± 56 vs. 228 ± 52 × 10^9^/L; *p* = 0.18).

Correlation analyses between the hematological indices and clinical or echocardiographic parameters are presented in Table 3. NLR, RDW, and PLR were positively correlated with NT-proBNP levels, NYHA functional class, E/e′ ratio, and left atrial volume index. NLR and RDW were inversely correlated with estimated glomerular filtration rate, whereas the correlation between PLR and estimated glomerular filtration rate was not statistically significant. None of the three hematological indices was significantly correlated with systolic blood pressure.

The frequencies of the 12-month clinical outcomes according to biomarker tertiles are presented in Table 4. The proportions of patients experiencing MACEs, all-cause mortality, and heart failure-related rehospitalization increased across successive tertiles of NLR, with statistically significant trends for all three outcomes. Similar increases across tertiles were observed for RDW and PLR. The corresponding *p*-values for trend are reported in Table 4.

**Figure 1:**
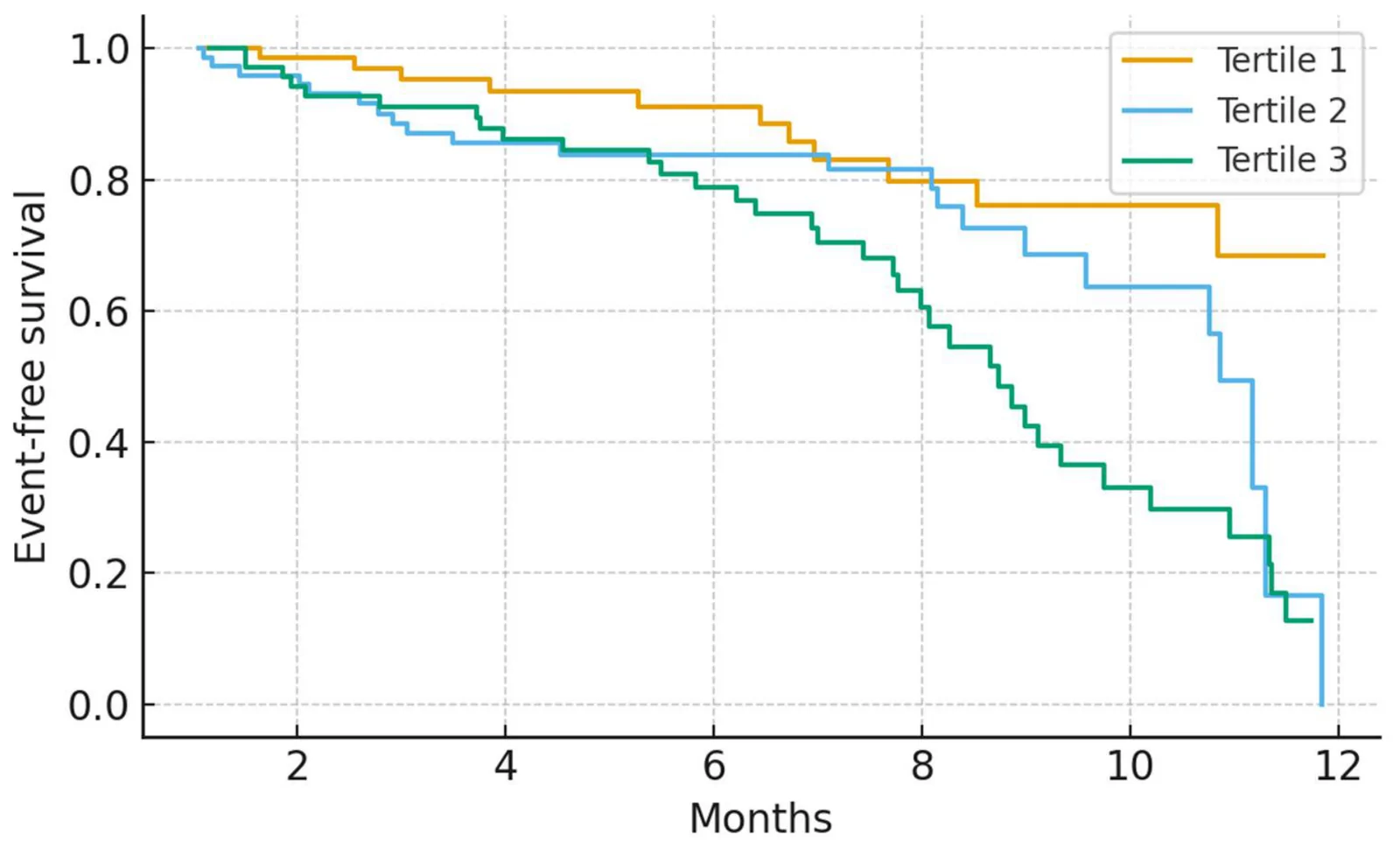
Kaplan–Meier curves for MACE-free survival according to NLR tertiles.

**Figure 2:**
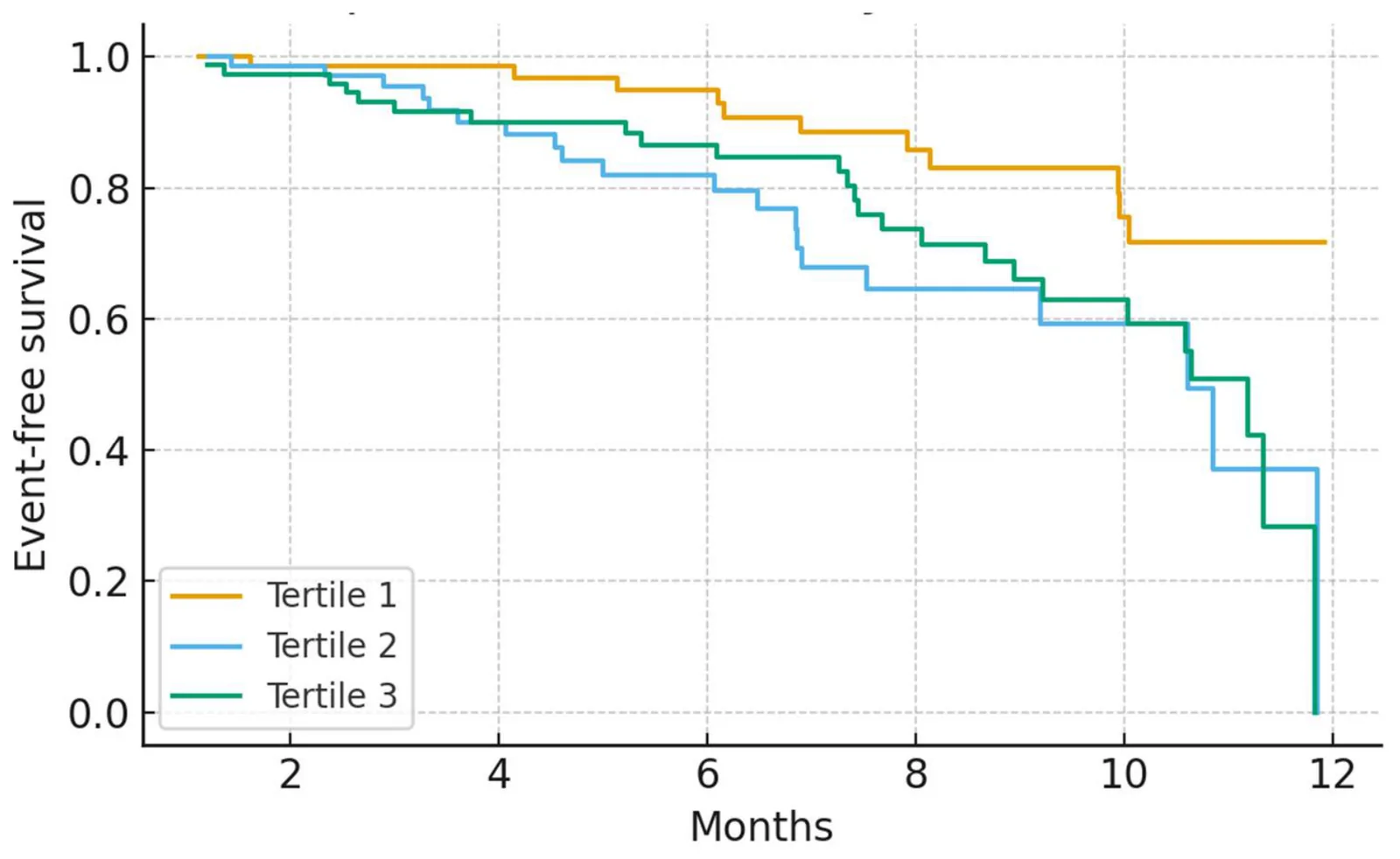
Kaplan–Meier curves for MACE-free survival according to RDW tertiles.

**Figure 3:**
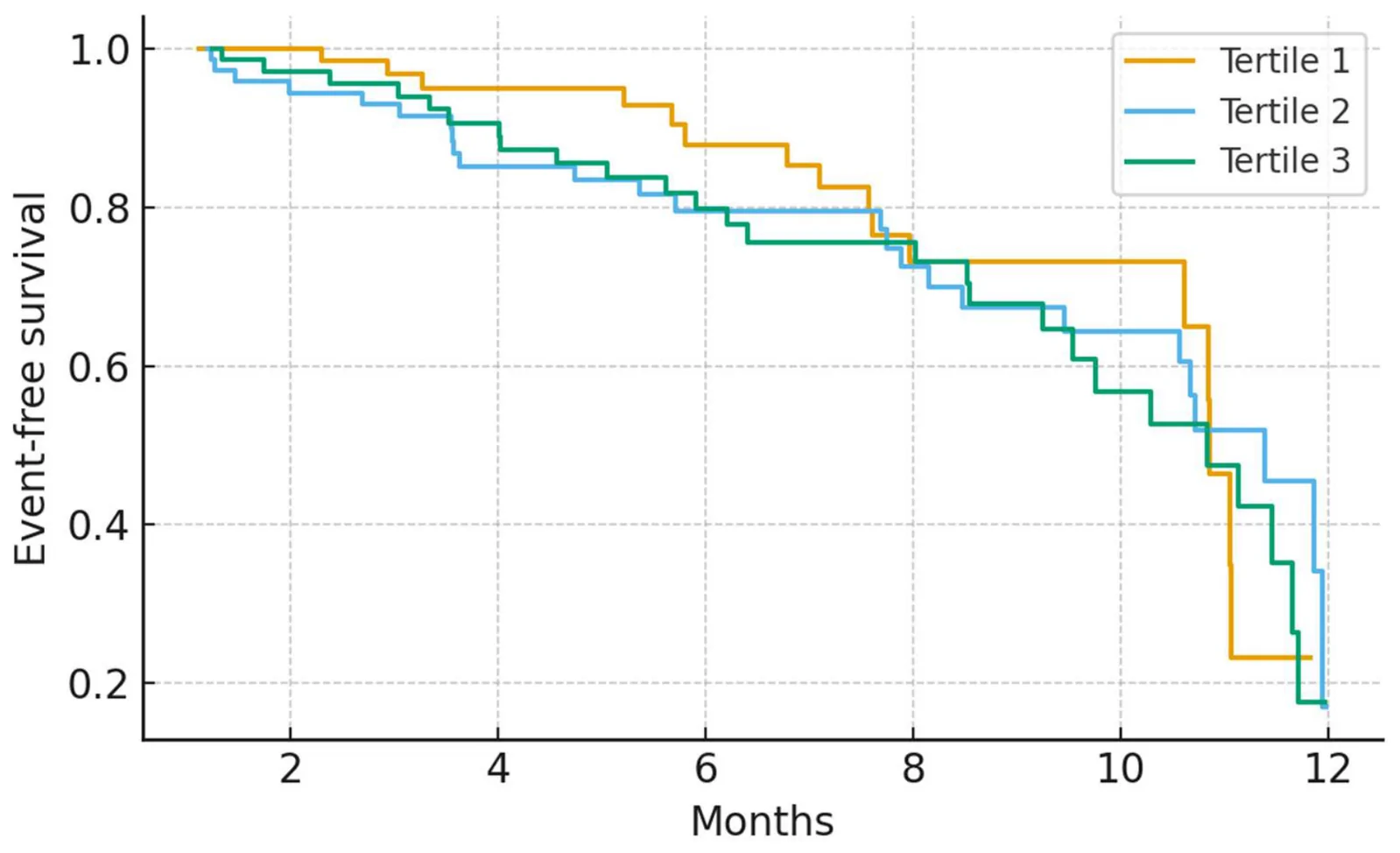
Kaplan–Meier curves for MACE-free survival according to PLR tertiles.

Kaplan–Meier analyses showed differences in MACE-free survival across tertiles of NLR, RDW, and PLR (Figure 1, Figure 2 and Figure 3). For each hematological index, patients in the highest tertile had the lowest MACE-free survival during follow-up, those in the lowest tertile had the highest MACE-free survival, and the intermediate tertile showed an intermediate survival trajectory.

**Figure 4:**
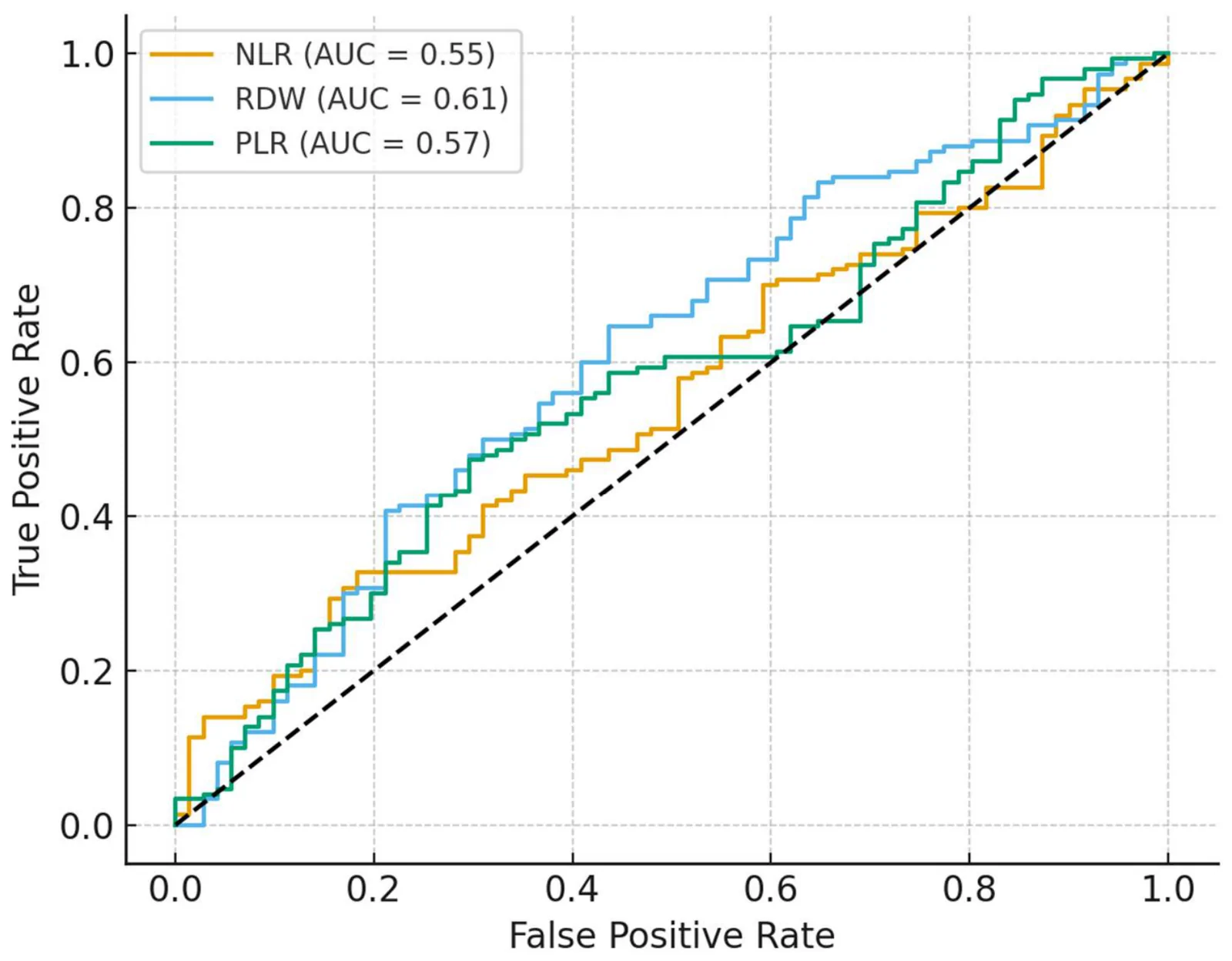
Receiver operating characteristic (ROC) curves for predicting 12-month MACEs using NLR, RDW, and PLR.

ROC curve analysis demonstrated limited ability of the three hematological indices to discriminate between patients with and without 12-month MACEs (Figure 4). AUC values ranged from approximately 0.55 to 0.61, with RDW showing the highest, although still modest, discrimination. NLR and PLR had AUC values closer to 0.50. Therefore, none of the three indices demonstrated adequate discriminative performance when considered individually. These findings should be distinguished from the Cox regression results, which indicate statistical associations with MACEs after adjustment but do not establish sufficient accuracy for individual risk classification. The ability of the hematological indices to discriminate between patients who did and did not experience 12-month MACEs was evaluated using ROC curve analysis (Figure 4). Discrimination was limited for all three indices, with AUC values ranging from approximately 0.55 to 0.61. RDW showed the highest AUC, followed by NLR and PLR; however, none of the markers demonstrated sufficient accuracy to support use as an individual prognostic test. The incremental predictive value of these indices beyond established clinical, biochemical, and echocardiographic models was not evaluated.

In the multivariable Cox proportional hazards model, higher NLR and RDW values remained independently associated with an increased risk of 12-month MACEs after simultaneous adjustment for demographic characteristics, major comorbidities, NT-proBNP, and the E/e′ ratio (Table 5). Each one-unit increase in NLR was associated with a 28% higher hazard of MACEs (HR 1.28, 95% CI 1.11–1.48; *p* = 0.001), while each one-percentage-point increase in RDW was associated with a 32% higher hazard (HR 1.32, 95% CI 1.14–1.54; *p* < 0.001). PLR showed only a borderline association and did not reach statistical significance (HR 1.05 per 10-unit increase, 95% CI 0.99–1.11; *p* = 0.08). NT-proBNP and the E/e′ ratio also remained independently associated with MACEs, whereas age, sex, hypertension, type 2 diabetes mellitus, and chronic kidney disease were not statistically significant in the adjusted model.

## 4. Discussion

In this retrospective study involving both inpatients and outpatients with confirmed HFpEF, we observed that hematological inflammatory indices derived from the complete blood count-namely NLR, RDW, and PLR, were significantly higher in patients with HFpEF compared with individuals without heart failure and demonstrated meaningful associations with key markers of disease severity. Moreover, NLR and RDW emerged as independent predictors of 12-month MACEs after multivariable adjustment for age, sex, comorbidities, NT-proBNP, and echocardiographic parameters. These findings reinforce the growing body of evidence indicating that systemic inflammation and impaired cellular homeostasis contribute substantially to the pathophysiology, clinical progression, and prognosis of HFpEF [23,24].

The observation that patients with HFpEF exhibit higher NLR values compared with controls aligns with multiple studies describing the inflammatory character of HFpEF [25]. Neutrophils, as first-line responders in innate immunity, release proteolytic enzymes, reactive oxygen species, and proinflammatory cytokines, contributing to endothelial dysfunction and microvascular inflammation [26]. Lymphopenia, the denominator of the NLR, reflects chronic sympathetic activation, neurohormonal stress, and immunosenescence, processes commonly amplified in HFpEF [27]. Prior work by Horne et al. and more recent HFpEF cohorts reported NLR thresholds between 3 and 4 being associated with adverse outcomes, consistent with our tertile-based gradient of risk [13]. From a physiopathological perspective, elevated NLR mirrors a systemic inflammatory milieu that promotes stiffening of the myocardium through endothelial activation, reduced nitric oxide bioavailability, and increased myocardial fibrosis [28].

RDW, a measure of anisocytosis, was associated with markers of disease severity and adverse outcomes in our cohort. However, RDW is a nonspecific hematological parameter influenced by several processes, including inflammation, oxidative stress, renal dysfunction, nutritional deficiencies, iron-restricted erythropoiesis, and anemia [29]. In the present study, hemoglobin was modestly but significantly lower in patients with HFpEF than in controls (12.6 ± 1.2 vs. 12.9 ± 1.1 g/dL; *p* = 0.04), raising the possibility that part of the observed RDW signal reflected differences in anemia or iron status rather than inflammation alone. Tkaczyszyn et al. demonstrated that iron parameters remained independently associated with RDW and other red-cell indices in patients with heart failure and that iron deficiency was common even in the absence of anemia or overt abnormalities in red-cell indices [30]. Similarly, an analysis of patients with HFpEF from PARAGON-HF reported an association between higher RDW and lower hemoglobin, while acknowledging that iron status was not available [31]. Because ferritin, transferrin saturation, and other iron parameters were not measured in our cohort, we could not determine the relative contributions of inflammation, anemia, and iron deficiency to the observed RDW associations. RDW should therefore be interpreted as an integrative but nonspecific marker of altered erythropoiesis and systemic disease burden rather than as a marker of inflammation alone [32,33,34].

PLR showed more modest associations with disease severity and a weaker prognostic value after adjustment. This is consistent with the literature, where PLR, although indicative of platelet activation and lymphocyte suppression, tends to have less prognostic power than RDW or NLR in heart failure populations [35]. Platelet activation contributes to microvascular thrombosis and endothelial dysfunction, both implicated in HFpEF pathogenesis, but PLR appears to be a less precise integrator of these processes [18]. Nonetheless, its significance in univariate analyses indicate that platelet-related inflammation and thrombogenicity play a role, albeit secondary, in shaping the HFpEF inflammatory profile.

Our correlation analyses strengthen the conceptual link between these hematologic indices and HFpEF pathophysiology. The positive correlations between NLR and markers of congestion such as NT-proBNP and E/e’ point to a relationship between systemic inflammation and hemodynamic compromise. Chronic inflammatory signaling has been shown to impair endothelial nitric oxide synthase activity, reducing vasodilatory capacity and increasing ventricular filling pressures [36]. The enlargement of the left atrium, which correlated with both NLR and RDW in our study, is a structural hallmark of chronically elevated filling pressures and reinforces the interplay between inflammation, stiffness, and diastolic dysfunction [37,38,39].

The analyses showed graded associations between higher NLR and RDW tertiles and adverse clinical outcomes. However, these associations should be distinguished from predictive performance. Although the Kaplan–Meier curves demonstrated differences in MACE-free survival across tertiles, the ROC analysis showed only modest discrimination, with AUC values of approximately 0.55–0.61. Therefore, the present findings indicate an association with outcome risk but do not establish sufficient accuracy for individual prognostic classification. The observed associations are consistent with the paradigm that HFpEF is not simply a myocardial disease but a systemic disorder arising from multiorgan inflammation [40,41,42]. Increased NLR and RDW likely represent downstream manifestations of these processes. Inflammatory cytokines such as IL-6 and TNF-α, elevated in HFpEF, induce changes in bone marrow function, promote erythrocyte heterogeneity, and stimulate neutrophil proliferation, providing a coherent biological explanation for the hematological patterns observed in our cohort [43].

NLR and RDW remained statistically associated with MACEs after adjustment for age, comorbidities, NT-proBNP, and echocardiographic indices. Nevertheless, an independent association in a Cox model does not necessarily imply clinically meaningful discrimination or incremental predictive value. Because the study did not compare the performance of clinical models with and without these indices, their added prognostic value beyond established clinical, biochemical, and echocardiographic parameters remains uncertain [44]. The persistence of NLR and RDW as independent predictors suggests that inflammatory mechanisms may contribute to risk in HFpEF beyond hemodynamic impairment alone [45]. This observation supports the increasingly accepted concept that HFpEF is characterized by a “meta-inflammatory” state, combining cardiometabolic stress, impaired mitochondrial function, endothelial inflammation, and immunological dysregulation [46].

Importantly, the independent associations observed in the adjusted Cox model should not be interpreted as evidence of strong individual predictive performance. Hazard ratios quantify associations with the rate of an outcome, whereas the AUC evaluates the ability of a marker to distinguish between patients who do and do not experience that outcome. In the present study, the AUC values of approximately 0.55–0.61 indicate that NLR, RDW, and PLR offered only limited discrimination when used individually. Therefore, although NLR and RDW remained statistically associated with MACEs after adjustment, their clinical utility for individual risk prediction remains uncertain.

When interpreted in the anatomical and physiological context, these findings become even more meaningful. HFpEF is characterized by concentric remodeling, increased ventricular and vascular stiffness, and diffuse microvascular rarefaction [47]. These processes are strongly modulated by inflammation [48]. Neutrophil-mediated oxidative stress can directly damage endothelial layers, while cytokine-mediated signaling promotes fibroblast activation and collagen deposition, leading to myocardial stiffening [49]. RDW elevation may reflect impaired bone marrow response to chronic cardiac and systemic stress, mirroring the reduced physiologic reserve characteristic of HFpEF patients [50]. Clinically, this translates to reduced exercise capacity, increased susceptibility to decompensation, and higher long-term mortality, patterns clearly illustrated by the outcome differences observed across biomarker tertiles.

Additionally, the modest but significant correlations between these indices and renal function parameters highlight the bidirectional interaction between the heart and kidneys in HFpEF [51]. Reduced eGFR reflects both renal congestion and intrinsic renal inflammation, processes that can exacerbate systemic inflammatory activity and thereby elevate NLR and RDW [52]. Although adjustments for renal function attenuated some associations, they did not fully eliminate the prognostic significance of these indices, suggesting that the inflammatory signal captured by NLR and RDW is not merely a surrogate for renal impairment.

### Future Directions, Strengths and Limitations

Future research should validate the prognostic value of NLR, RDW, and PLR in larger, multicenter and prospective cohorts. Serial measurements could clarify whether longitudinal changes in these indices better reflect congestion, inflammatory activity, or therapeutic response. Integrating these simple hematologic markers with advanced phenotyping, such as metabolomics, endothelial function testing, or cardiac MRI, may help elucidate the mechanistic pathways that link inflammation, microvascular dysfunction, and myocardial stiffness in HFpEF. Future studies should determine whether NLR or RDW provides incremental value beyond established clinical prediction models by using formal model comparisons, calibration analyses, reclassification metrics, and external validation.

This study benefits from a real-world cohort that includes both ambulatory and hospitalized patients, reflecting the broad clinical spectrum of HFpEF. The control group was drawn from the same institution and study period as the HFpEF cohort. Although the two groups showed broadly similar distributions of age and major cardiovascular risk factors, no formal matching procedure was performed. Hematologic indices were derived from routine CBC testing, making the findings highly applicable in everyday clinical practice. The analysis included clinically meaningful endpoints at 12 months and used multivariable Cox models adjusted for demographic, biochemical, and echocardiographic parameters, ensuring robustness of the prognostic results.

The retrospective design limits causal interpretation, and residual confounding from unmeasured inflammatory or metabolic factors may persist. Hematologic indices were evaluated at a single time point, preventing assessment of temporal trends. Echocardiographic measurements were obtained from clinical practice rather than standardized research protocols, introducing variability. Biomarkers of inflammation (e.g., CRP, IL-6) were not available, limiting mechanistic interpretation. Finally, this single-center study may limit generalizability, and validation in diverse populations is needed. Furthermore, because the control group was not formally matched to the HFpEF cohort, selection bias and residual confounding related to baseline differences between the groups cannot be excluded.

Baseline pharmacological treatment may also have influenced the observed 12-month outcomes. Diuretic use may partly reflect a greater burden of congestion, whereas treatment with renin–angiotensin system inhibitors, mineralocorticoid receptor antagonists, beta-blockers, or SGLT2 inhibitors may modify the risk of cardiovascular events and heart failure-related hospitalization. Because treatment was not randomly assigned, medication use may represent both a therapeutic exposure and a marker of disease severity or comorbidity burden. Medication classes were not incorporated into the primary Cox model because the limited number of MACEs did not support the inclusion of several additional covariates without increasing the risk of overfitting. Furthermore, information regarding medication dose, treatment duration, adherence, and changes during follow-up was not consistently available. Residual confounding related to baseline and subsequent treatment therefore cannot be excluded. No diagnostic ROC analysis was performed to assess the ability of these indices to distinguish HFpEF from individuals without heart failure. Therefore, the observed between-group differences should not be interpreted as evidence of diagnostic utility. Furthermore, the prognostic ROC analyses showed only modest discrimination, and no formal assessment of incremental predictive value beyond established clinical models was performed.

Iron status was not assessed because ferritin, serum iron, transferrin saturation, and total iron-binding capacity were not consistently available. Given the modestly lower hemoglobin concentration in the HFpEF group and the established association of RDW with iron-restricted erythropoiesis, part of the RDW signal may have reflected anemia or iron deficiency rather than systemic inflammation alone. This limits the mechanistic interpretation of RDW and should be addressed in future studies incorporating comprehensive iron-status assessment.

## 5. Conclusions

In this single-center retrospective study, NLR, RDW, and PLR were significantly higher in patients with HFpEF than in individuals without heart failure and were associated with clinical, biochemical, and echocardiographic markers of disease severity. Within the HFpEF cohort, higher NLR and RDW values were associated with increased rates of adverse outcomes, and both indices remained independently associated with 12-month MACEs after multivariable adjustment. However, all three indices demonstrated limited individual discrimination in the ROC analysis, with AUC values of approximately 0.55–0.61. Therefore, the independent associations observed in the Cox model should not be interpreted as evidence of strong individual predictive performance. Moreover, because no diagnostic ROC analysis was performed, the observed differences between patients with HFpEF and controls do not establish diagnostic utility. NLR, RDW, and PLR should not be used as standalone diagnostic or prognostic tools. Rather, they should be regarded as readily available but nonspecific hematological markers reflecting multiple underlying processes, including systemic inflammation, comorbidity burden, altered erythropoiesis, anemia, and potentially iron deficiency. Their incremental value beyond established clinical, biochemical, and echocardiographic models requires confirmation through formal model-comparison analyses and external validation in larger prospective cohorts.

## Figures and Tables

**Table 1:** Baseline demographic, clinical, and echocardiographic characteristics of the study population.

Characteristic	HFpEF (n = 221)	Control (n = 143)	*p*-Value
Age, years (mean ± SD)	70.8 ± 8.5	69.1 ± 9.1	0.18
Female sex (%)	60.6%	58.0%	0.63
BMI (kg/m^2^)	30.4 ± 5.1	29.6 ± 4.8	0.21
Hypertension (%)	82.4%	76.2%	0.19
Type 2 diabetes mellitus (%)	38.5%	32.9%	0.24
Dyslipidemia (%)	62.0%	57.3%	0.49
Chronic kidney disease (%)	24.0%	18.9%	0.26
Atrial fibrillation (%)	29.9%	22.4%	0.08
NYHA class III-IV (%)	39.4%	-	-
Systolic blood pressure (mmHg)	144 ± 17	141 ± 16	0.12
Heart rate (bpm)	77 ± 11	75 ± 10	0.09
Left ventricular ejection fraction (%)	58.5 ± 4.0	59.1 ± 3.7	0.16
E/e’ ratio	15.5 ± 4.0	10.1 ± 2.3	<0.001
Left atrial volume index (mL/m^2^)	41.9 ± 10.8	30.4 ± 7.6	<0.001
NT-proBNP (pg/mL), median (IQR)	820 (510–1410)	165 (82–280)	<0.001
Creatinine (mg/dL)	1.18 ± 0.30	1.10 ± 0.27	0.06
Baseline treatment in the HFpEF cohort			
Diuretics, n (%)	158 (71.5%)	-	
Beta-blockers, n (%)	139 (62.9%)	-	
ACEI or ARB, n (%)	116 (52.5%)	-	
ARNI, n (%)	22 (10.0%)	-	
Mineralocorticoid receptor antagonists, n (%)	72 (32.6%)	-	
SGLT2 inhibitors, n (%)	51 (23.1%)	-	
Other glucose-lowering medications, n (%)	66 (29.9%)	-	

ACEI, angiotensin-converting enzyme inhibitor; ARB, angiotensin receptor blocker; ARNI, angiotensin receptor–neprilysin inhibitor; SGLT2, sodium–glucose cotransporter 2. Medication categories were not mutually exclusive. Baseline treatment was reported for the HFpEF cohort because the 12-month prognostic analyses were restricted to these patients.

**Table 2:** Comparison of hematological inflammatory indices between HFpEF and control groups.

Hematological Index	HFpEF (n = 221)	Control (n = 143)	*p*-Value
NLR	3.98 ± 0.98	2.72 ± 0.81	<0.001
RDW (%), Red Cell Distribution Width	14.62 ± 1.09	13.48 ± 0.92	<0.001
PLR (Platelet-to-Lymphocyte Ratio)	152.4 ± 38.7	131.9 ± 29.6	<0.001
Hemoglobin (g/dL)	12.6 ± 1.2	12.9 ± 1.1	0.04
Platelet count (×10^9^/L)	235 ± 56	228 ± 52	0.18

**Table 3:** Hematological inflammatory indices and clinical/severity markers in the HFpEF group.

Severity Marker	NLR (r, *p*-Value)	RDW (%) (r, *p*-Value)	PLR (r, *p*-Value)
NT-proBNP (pg/mL)	r = 0.41, *p* < 0.001	r = 0.38, *p* < 0.001	r = 0.29, *p* < 0.001
NYHA class	r = 0.33, *p* < 0.001	r = 0.27, *p* < 0.001	r = 0.21, *p* = 0.002
E/e’ ratio	r = 0.36, *p* < 0.001	r = 0.31, *p* < 0.001	r = 0.19, *p* = 0.005
Left atrial volume index (LAVI)	r = 0.28, *p* < 0.001	r = 0.25, *p* < 0.001	r = 0.14, *p* = 0.04
Estimated glomerular filtration rate (eGFR)	r = −0.24, *p* < 0.001	r = −0.30, *p* < 0.001	r = −0.12, *p* = 0.08
Systolic blood pressure (SBP)	r = 0.09, *p* = 0.18	r = 0.06, *p* = 0.32	r = 0.05, *p* = 0.41

**Table 4:** Twelve-month clinical outcomes according to tertiles of NLR, RDW, and PLR in the HFpEF cohort.

Biomarker/Tertile	N	MACEs, n (%)	All-Cause Mortality, n (%)	HF Rehospitalization, n (%)
NLR				
T1 ≤ 3.20	74	11 (14.9%)	5 (6.8%)	14 (18.9%)
T2 3.21–4.30	73	18 (24.7%)	9 (12.3%)	21 (28.8%)
T3 ≥ 4.31	74	33 (44.6%)	16 (21.6%)	33 (44.6%)
Total	221	62 (28.1%)	30 (13.6%)	68 (30.8%)
*p*-trend	—	<0.001	0.008	<0.001
RDW				
T1 ≤ 13.9%	74	10 (13.5%)	4 (5.4%)	13 (17.6%)
T2 14.0–15.4%	73	19 (26.0%)	8 (11.0%)	20 (27.4%)
T3 ≥ 15.5%	74	33 (44.6%)	18 (24.3%)	35 (47.3%)
Total	221	62 (28.1%)	30 (13.6%)	68 (30.8%)
*p*-trend	—	<0.001	<0.001	<0.001
PLR				
T1 ≤ 128	74	14 (18.9%)	6 (8.1%)	15 (20.3%)
T2 129–158	73	20 (27.4%)	9 (12.3%)	22 (30.1%)
T3 ≥ 159	74	28 (37.8%)	15 (20.3%)	31 (41.9%)
Total	221	62 (28.1%)	30 (13.6%)	68 (30.8%)
*p*-trend	—	0.010	0.031	0.004

Data are presented as the number and percentage of patients who experienced at least one qualifying event during the 12-month follow-up. MACEs were defined as a composite of cardiovascular death, non-fatal myocardial infarction, or non-fatal stroke. Heart failure-related rehospitalization was analyzed as a separate outcome and was not included in the MACE composite. The overall numbers of events were 62 MACEs, 30 all-cause deaths, and 68 heart failure-related rehospitalizations in each biomarker stratification. *p*-values for trend were calculated across ordered tertiles.

**Table 5:** Multivariable Cox proportional hazards model for predicting 12-month MACEs in the HFpEF cohort.

Variable	Hazard Ratio (HR)	95% Confidence Interval	*p*-Value
NLR (per unit increase)	1.28	1.11–1.48	0.001
RDW (% per 1-unit increase)	1.32	1.14–1.54	<0.001
PLR (per 10-unit increase)	1.05	0.99–1.11	0.08
Age (per 1 year)	1.02	0.99–1.04	0.12
Female sex	0.91	0.61–1.35	0.63
Hypertension	1.09	0.71–1.67	0.68
Type 2 diabetes mellitus	1.21	0.83–1.76	0.31
Chronic kidney disease	1.27	0.87–1.86	0.21
NT-proBNP (per 100 pg/mL)	1.08	1.03–1.14	0.004
E/e’ ratio (per 1-unit increase)	1.06	1.02–1.10	0.002

Hazard ratios for NLR are reported per 1-unit increase, and those for RDW per 1-percentage-point increase. PLR is reported per 10-unit increase because PLR values are numerically larger and a 1-unit change would yield a hazard ratio very close to 1, making clinical interpretation less intuitive. This rescaling affects the numerical expression of the hazard ratio but does not alter the statistical significance or overall fit of the model.

## Data Availability

The data supporting the findings of this study are available from the corresponding author upon reasonable request. Due to institutional data protection policies of the participating hospital, the datasets are not publicly available.
